# Congestive Heart Failure Leads to Prolongation of the PR Interval and Atrioventricular Junction Enlargement and Ion Channel Remodelling in the Rabbit

**DOI:** 10.1371/journal.pone.0141452

**Published:** 2015-10-28

**Authors:** Theodora Nikolaidou, Xue J. Cai, Robert S. Stephenson, Joseph Yanni, Tristan Lowe, Andrew J. Atkinson, Caroline B. Jones, Rida Sardar, Antonio F. Corno, Halina Dobrzynski, Philip J. Withers, Jonathan C. Jarvis, George Hart, Mark R. Boyett

**Affiliations:** 1 Institute of Cardiovascular Sciences, University of Manchester, Manchester, United Kingdom; 2 School of Sport and Exercise Sciences, Liverpool John Moores University, Liverpool, United Kingdom; 3 The Manchester Henry Moseley X-ray Imaging Facility, University of Manchester, Manchester, United Kingdom; 4 School of Medical Sciences, Health Campus, University Sains Malaysia, Kubang Kerian, Malaysia; Gent University, BELGIUM

## Abstract

Heart failure is a major killer worldwide. Atrioventricular conduction block is common in heart failure; it is associated with worse outcomes and can lead to syncope and bradycardic death. We examine the effect of heart failure on anatomical and ion channel remodelling in the rabbit atrioventricular junction (AVJ). Heart failure was induced in New Zealand rabbits by disruption of the aortic valve and banding of the abdominal aorta resulting in volume and pressure overload. Laser micro-dissection and real-time polymerase chain reaction (RT-PCR) were employed to investigate the effects of heart failure on ion channel remodelling in four regions of the rabbit AVJ and in septal tissues. Investigation of the AVJ anatomy was performed using micro-computed tomography (micro-CT). Heart failure animals developed first degree heart block. Heart failure caused ventricular myocardial volume increase with a 35% elongation of the AVJ. There was downregulation of HCN1 and Cx43 mRNA transcripts across all regions and downregulation of Ca_v_1.3 in the transitional tissue. Cx40 mRNA was significantly downregulated in the atrial septum and AVJ tissues but not in the ventricular septum. mRNA abundance for ANP, CLCN2 and Na_v_β1 was increased with heart failure; Na_v_1.1 was increased in the inferior nodal extension/compact node area. Heart failure in the rabbit leads to prolongation of the PR interval and this is accompanied by downregulation of HCN1, Ca_v_1.3, Cx40 and Cx43 mRNAs and anatomical enlargement of the entire heart and AVJ.

## Introduction

Electrical conduction abnormalities are common in heart failure and lead to cardiac arrhythmias and sudden cardiac death [1]. The electrocardiographic PR interval represents conduction from the sinus node to the Purkinje fibres. It primarily reflects atrioventricular conduction [2]. Both in ischaemic and non-ischaemic cardiomyopathy first degree heart block is common [3] and approximately half of arrhythmic deaths are bradycardic in origin, including those due to atrioventricular block [4]. First degree atrioventricular block is associated with worse outcomes not only in patients with heart failure [3,5] but also in the general population [6]. Preserving atrioventricular synchrony is important in successful device therapy, including cardiac resynchronisation therapy (CRT). Patients with first degree atrioventricular block derive greater benefit from CRT [5].

Sinoatrial node (SAN) dysfunction in heart failure is associated with increased cycle length and prolongation of sinus node recovery time [7]. This is due to downregulation of HCN channels and reduction in *I*_f_ density [8–10]. Studies of atrial pathology in heart failure have shown atrial enlargement, scarring and impaired atrial conduction [11]. In animal models this is associated with a decrease in atrial *I*_Ca,L_, *I*_to_ and *I*_K,s_ [12]. In heart failure, left ventricular anatomical remodelling is associated with slowed contraction and relaxation, along with prolongation of the QRS duration. Peak *I*_Na_ is reduced with an increase in the late component *I*_Na,L_ [13]. Repolarizing K^+^ currents are reduced (*I*_to_, *I*_K,s_ and *I*_K,1_) [14] and Ca^2+^ handling is impaired [15].

The effect of heart failure on the physiology of the AVJ is unknown. The AVJ comprises the inferior nodal extension, compact node, and penetrating and His bundles and is surrounded by transitional tissue, which exhibits mixed atrial and atrioventricular characteristics [16]. The AVJ exhibits dual pathway physiology [17], with longitudinal dissociation of Cx43 expression in the inferior nodal extension [18]. The upper nodal bundle is devoid of Cx43 and is in continuity with the compact node, while the lower nodal bundle has small amounts of Cx43 and merges into the penetrating bundle [18]. Noujaim et al. [2] analysed the PR interval in 33 species and found that the PR interval changes by a single order of magnitude when the body mass changes by ≈6. We present here a study of the structural and molecular changes in the rabbit AVJ associated with congestive heart failure. Anatomical and physiological similarities between the rabbit and the human AVJ make this a relevant model [19].

## Methods

The primary objective of this study was to describe heart failure-induced mRNA changes in the rabbit AVJ and adjacent atrial and ventricular septum. The estimated effect size and level of variability informed by previous studies [20] were used to calculate the sample size. Gene expression analysis was performed in nine control and nine heart failure animals with laser microdissection and RT-PCR. mRNA was extracted from seven regions for each rabbit. RT-PCR results were analysed using 2-way ANOVA and the Limma test. Secondary objectives were the two- and three- dimensional reconstruction of the AVJ in heart failure and assessment of Cx43 protein expression. Two-dimensional reconstruction was performed in the same group of 18 animals using Masson’s trichrome and neurofilament immunostaining (S1 File). In a subset of this group (five control and six heart failure animals) immunohistochemistry was used to quantify Cx43 protein expression (S1 File). A separate sample of the same experimental groups (five control and five heart failure hearts) were processed for micro-CT imaging, as previously described [21]. Three-dimensional reconstruction and quantification of the total ventricular myocardial volume and AVJ volume and length were performed.

### Experimental animals

Animal procedures were undertaken in strict accordance with the United Kingdom Animals (Scientific Procedures) Act 1986 and were approved by the Ethical Review Process of the University of Manchester (project licence 40/3689). Three month old male New Zealand rabbits were purchased from an external breeding facility and housed singly in large cages in a standard animal holding facility. Environmental conditions were 12-hour light/dark cycle with food available from start of the light cycle and water *ad libitum*. Environmental enrichment included BBC channel 3 during the light cycle. Straw bedding was used. To allow adaptation to the environment and handling/feeding routine, animals were kept in the facility for 1–2 weeks prior to experimental use. Inspection and handling was performed at least twice daily and rabbits were weighed every 1–2 days. Echocardiography was performed in conscious animals before the first operation and approximately two-weekly subsequently. Allocation to experimental groups was entirely arbitrary. Animals were taken sequentially from adjacent cages and were numbered on arrival in theatre. No blinding was used.

Animals (2.5–3.0 kg; mean age 12 weeks, range 10–14 weeks at first operation) underwent a two-stage procedure under general anaesthesia to induce heart failure as previously described [22]. 12-week old animals were selected because smaller animals would be at risk of more complications and an unacceptable mortality, while older animals would take significantly longer to develop signs of heart failure. Operations took place between 8am and 5pm at the University Biomedical Services Facility operating theatres. Firstly, volume overload was induced by disrupting the aortic valve by repeated introduction of a catheter (external diameter 1.22 mm) through the leaflets until pulse pressure increased by approximately 100% (week 0). Banding of the abdominal aorta was performed three weeks later (week 3) using a silver clip (internal diameter 2.42 mm) just above the left renal artery. This resulted in reduction in aortic diameter by approximately 50% at that level. Sham-operated animals had a catheter inserted through the right carotid artery without disrupting the aortic valve. Three weeks later laparotomy was performed. Rabbits were anaesthetised using ketamine intramuscular injection (25–50 mg/kg) and isoflurane inhalation 2% with O_2_ and given one dose of baytril 0.2 ml/kg and temgesic 0.05 mg/kg subcutaneously preoperatively. These are all routine agents for sedation, anaesthesia, antibiotic prophylaxis and analgesia respectively. Animals were sacrificed five weeks after the second operation (week 8) by intravenous injection of phenobarbital 0.5 ml/kg. No adverse events occurred during experimental procedures. Expected signs of heart failure (poverty of movement, tachypnoea, serous cavity effusions) occurred in the heart failure group only.

### Functional measurements

Transthoracic echocardiography was performed using a GE Vivid 3 ultrasound machine and a 5S transducer before and after surgery. Rabbits were held in the supine or lateral decubitus position. Two-dimensional images were obtained in the parasternal long axis view and colour Doppler was used to assess the aortic and mitral valves for regurgitation. Using M-mode, left ventricular internal diameter was measured in systole and diastole and fractional shortening calculated. ECG recordings were performed in anaesthetised rabbits prior to the first surgical procedure and then again just before the animals were sacrificed. Custom-made pin electrodes were inserted subcutaneously in the region of the right and left forelimbs and left hindlimb. 1 mg of atropine was given followed by 3 mg of propranolol over 5 min followed by another dose of 1 mg atropine. The signal was amplified and filtered (Neurolog, Digitimer, UK) before being recorded directly onto a computer using Lab Chart Pro Software (AD Instruments, Australia). The signal was sampled at 1000 Hz. Baseline ECG was recorded for 10 min. Immediately following injection of atropine and propranolol a recording of ~2000 consecutive beats was taken.

### Micro-computed tomography

Histological sectioning followed by computer three-dimensional reconstruction has traditionally been considered the gold-standard in anatomic reconstruction of the AVJ [16]. Histology is limited by tissue disruption during sectioning and poor z-plane resolution and three-dimensional registration. The use of micro-CT for imaging the AVJ *ex vivo* has recently been demonstrated in rat, rabbit and dog hearts [21,23]. Five control and five heart failure rabbit hearts were prepared and stained with 3.75% I_2_KI for three days, as previously described.[21,23] Using the Nikon Metris Custom Bay 225/320 kV scanner at the Manchester X-ray Imaging Facility (MXIF, University of Manchester), samples were rotated about a vertical axis through 360° during which 1440–3500 frames (radiographs) were acquired. Imaging parameters were adjusted for each sample depending on size and staining to achieve optimal spatial and contrast resolution. Accordingly, the accelerating voltage was varied between 120–155 kV with overall scanning times varying between 20–60 min. Data was reconstructed using filter backprojection. Regions of interest (ROI) were segmented in Amira 5.33 software using a semi-automatic technique [21,23,24]. The resulting three-dimensional isosurfaces within the ROI were viewed in Amira 5.33 and their volumes calculated. Lengths were calculated using the ‘3D measure’ tool (Amira 5.4.0).

### Laser microdissection, RNA isolation and real-time polymerase chain reaction

AVJ tissues were sectioned at 50 μm thickness onto polyethylene naphthalate membrane slides. Laser microdissection (Leica LMD6000) was used to sample tissue from the atrial septum, transitional tissue, inferior nodal extension, compact node, penetrating bundle, His bundle and left and right ventricular septum. Each region was brought into focus at 6.3× magnification on approximately 50 serial sections, dissected and transported into a collection tube by gravity. Following laser microdissection, 20 μl RNA was extracted and purified using the RNAqueous-Micro kit (Ambion), which includes a DNase inactivation step. 160 ng of RNA were diluted to a total volume of 16 μl using RNAse free water. RNA quantity and quality was assessed using a Nanodrop 1000 spectrophotometer (Thermo Scientific) and by electrophoretic separation using an Agilent NanoLabChip [25]. This process took 1.5 h from thawing to mRNA extraction. First strand cDNA was synthesised by mixing 16 μl of mRNA with 4 μl High Capacity RNA-to-cDNA Master Mix Kit (Ambion, 4390779). The manufacturer’s thermal cycling schedule was used and aliquots of the resultant cDNA were diluted 1:10 for use in RT-PCR.

Reverse transcription was followed by RT-PCR of cDNA using the ABI Prism 7900HT system and SYBR Green in 10 μl reactions performed in triplicate. The endogenous control was the 28s ribosomal RNA. Primer sequences are available in S1 Table. For RT-PCR data baseline corrections and efficiency adjustments were made for each set of primers using the LinReg software [26]. CT values were corrected for efficiency by multiplying each CT value by the average efficiency for the set of primers. This adjusted CT values to what they would be if reaction conditions were ideal and the amount of cDNA doubled at each PCR cycle. RealTime StatMiner (Integromics) was used to identify technical outliers and biological outliers. Four out of 123 samples were excluded as biological outliers (with more than 20 flags using the MAD test). ΔCT values were calculated by ΔCT = 2^-[CT(sample)-CT(28s)]^.

### Statistical analysis

RT-PCR data are presented as means±SEM. 2-way ANOVA allows the simultaneous study of within-groups variance and between-groups variance. It was used to assess the effect of heart failure across multiple regions. Comparisons between control and heart failure in an individual region were made using the Limma test, which uses a moderated t-statistic, but, unlike the Student’s t-test standard errors are moderated across several genes [27]. Significance level was P<0.05 for 2-way ANOVA and P<0.05 for the FDR-adjusted Limma test using the Benjamini-Hochberg procedure. Other statistical analyses were performed using the two-tailed Student’s t-test.

## Results

### Prolongation of the PR interval in a rabbit heart failure model of hypertrophy and dilatation

**Fig 1:**
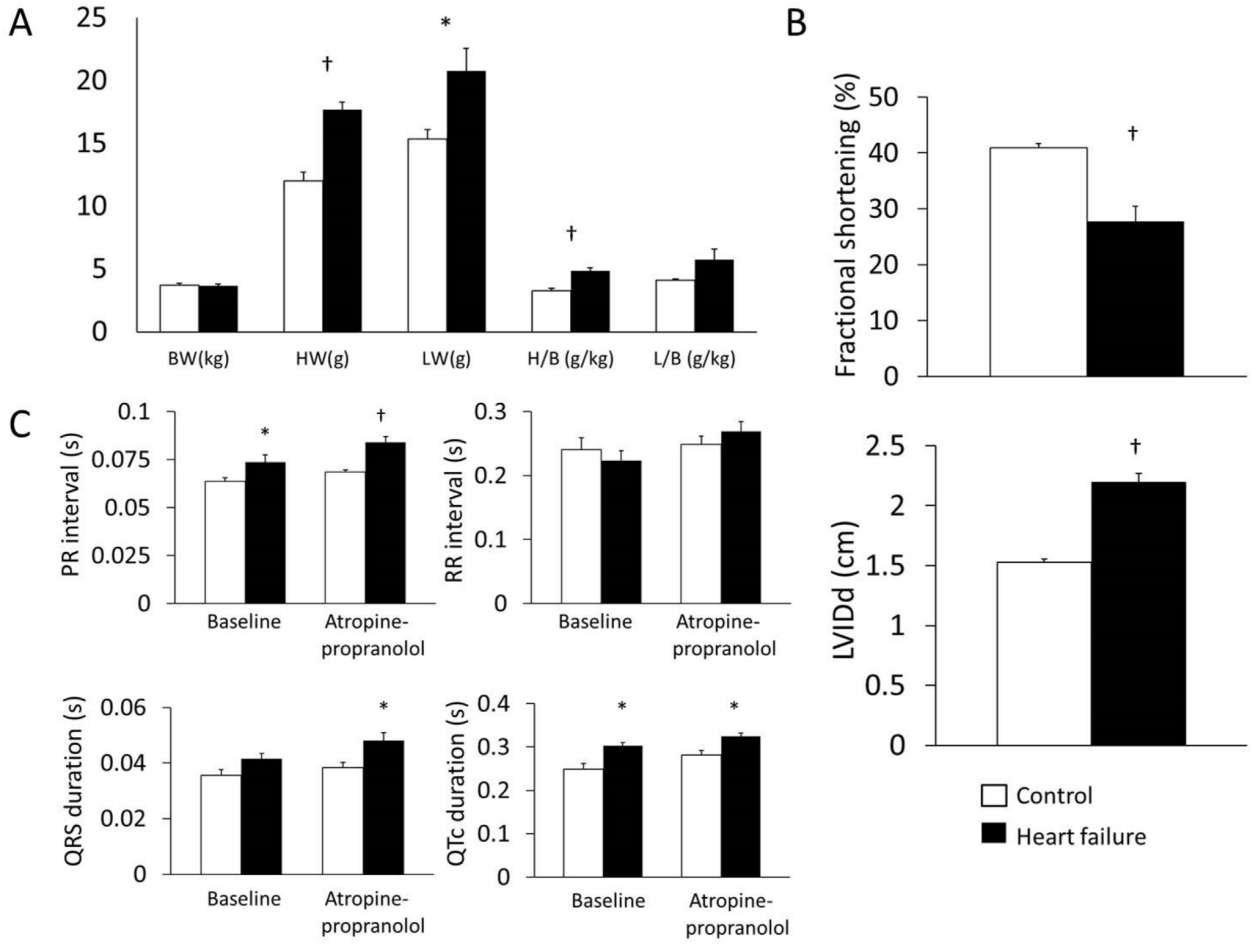
Rabbit model of heart failure. A, heart weight (HW), lung weight (LW), heart-to-body weight ratio (H/B) and lung-to-body weight ratio (L/B). B, echocardiography measurements. LVIDd, left ventricular internal diameter in end-diastole. C, ECG intervals before and after autonomic blockade. Means±SEM, *P<0.05, †P<0.001. White bars, control animals; black bars, heart failure animals.

Heart weight and heart-to-body weight ratio were increased in heart failure animals (heart weight, 12.0±0.70 g vs. 17.7±0.59 g, P = 1.4×10^−5^; heart-to-body weight ratio, 3.24±0.22 g/kg vs. 4.87±0.24 g/kg, P = 1.2×10^−4^; n = 9; Fig 1A). Lung weight was also increased in heart failure (P = 0.04; Fig 1A). Echocardiography confirmed reduced fractional shortening in heart failure (40.9±0.75% vs. 27.7±2.76%; n = 8; P = 3.9×10^−4^; Fig 1B). Left ventricular internal diameter in end-diastole increased (1.53±0.03 cm vs. 2.19±0.08 cm; n = 8; P = 1.1×10^−6^; Fig 1B). There was a tendency for heart rate to decrease after autonomic blockade in heart failure animals. The PR interval was longer in heart failure both at baseline and after autonomic blockade (baseline, 0.06±0.002 s vs. 0.07±0.003 s, P = 0.049; after autonomic blockade, 0.069±0.0009 s vs. 0.08±0.003 s; P = 6×10^−4^; n = 7/9; Fig 1C). QRS duration at baseline was 0.036±0.002 s for control vs. 0.042±0.002 s for heart failure animals (P = 0.06). After atropine and propranolol QRS duration was longer in heart failure (0.038±0.002 s vs. 0.048±0.003 s; P = 0.02; Fig 1C). QT duration was prolonged in heart failure (baseline, 0.123±0.01 s vs. 0.149±0.006 s, P = 0.05; after autonomic blockade, 0.140±0.008 s vs. 0.170±0.005 s, P = 0.007; Fig 1C). Similarly, QTc was 0.249±0.01 s for control vs. 0.302±0.007 s for heart failure animals at baseline (P = 0.003; Fig 1C) and 0.281±0.01 s for control vs. 0.324±0.008 s for heart failure after atropine and propranolol (P = 0.008; Fig 1C).

### Heart failure causes enlargement of the AVJ

**Fig 2:**
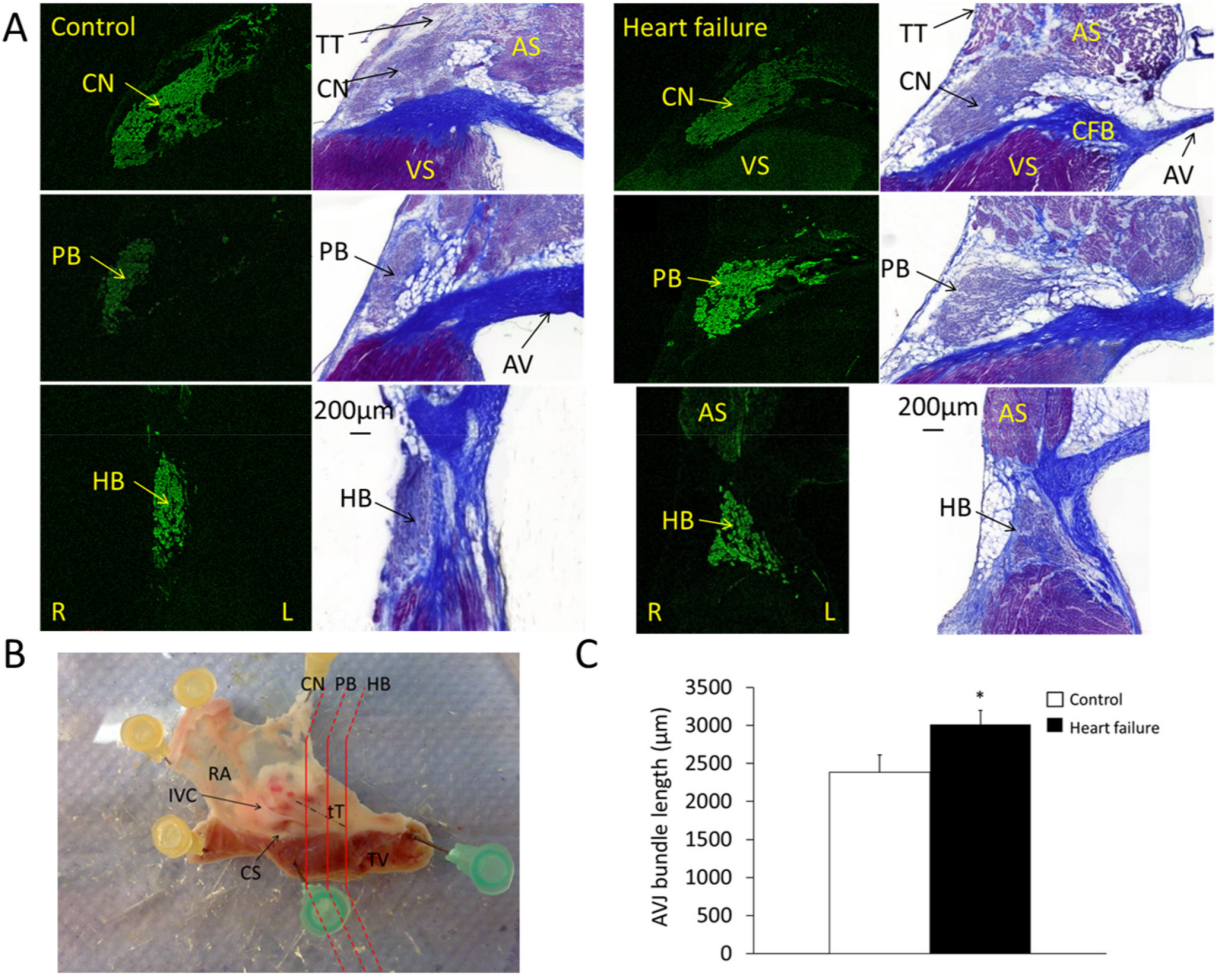
Histological reconstruction of the AVJ. A, representative staining of the AVJ for control and heart failure animals. The regions shown are compact node, penetrating bundle and His bundle. Neurofilament immunostaining (green signal) was performed for identification of the AVJ and the same section then stained with Masson’s trichrome for tissue characterisation (nuclei, blue-black; cytoplasm, red; collagen, blue). B, dissected AVJ sample as seen from the endocardium. Histological sectioning was performed along the planes shown by the red lines. C, atrioventricular bundle long axis measurement determined by virtual stacking of serial histological sections (means±SEM; *P<0.05, n = 9). AV, aortic valve; AS, atrial septum; CFB, central fibrous body; CN, compact node; HB, His bundle; L, left; PB, penetrating bundle; R, right; RA, right atrium; Tt, tendon of Todaro; VS, ventricular septum. White bars, control animals; black bars, heart failure animals.

Virtual stacking of serial histological sections in the coronal plane allowed measurement of AVJ length (Fig 2). For accuracy, AVJ bundle length measurements were made from the inferior border of the penetrating bundle (where it becomes fully insulated by fibrous tissue) to the branching point of the left bundle (atrioventricular bundle). Heart failure caused a 26% elongation of the atrioventricular bundle (2384±231 μm vs. 3014±186 μm; n = 9; P = 0.03).

**Fig 3:**
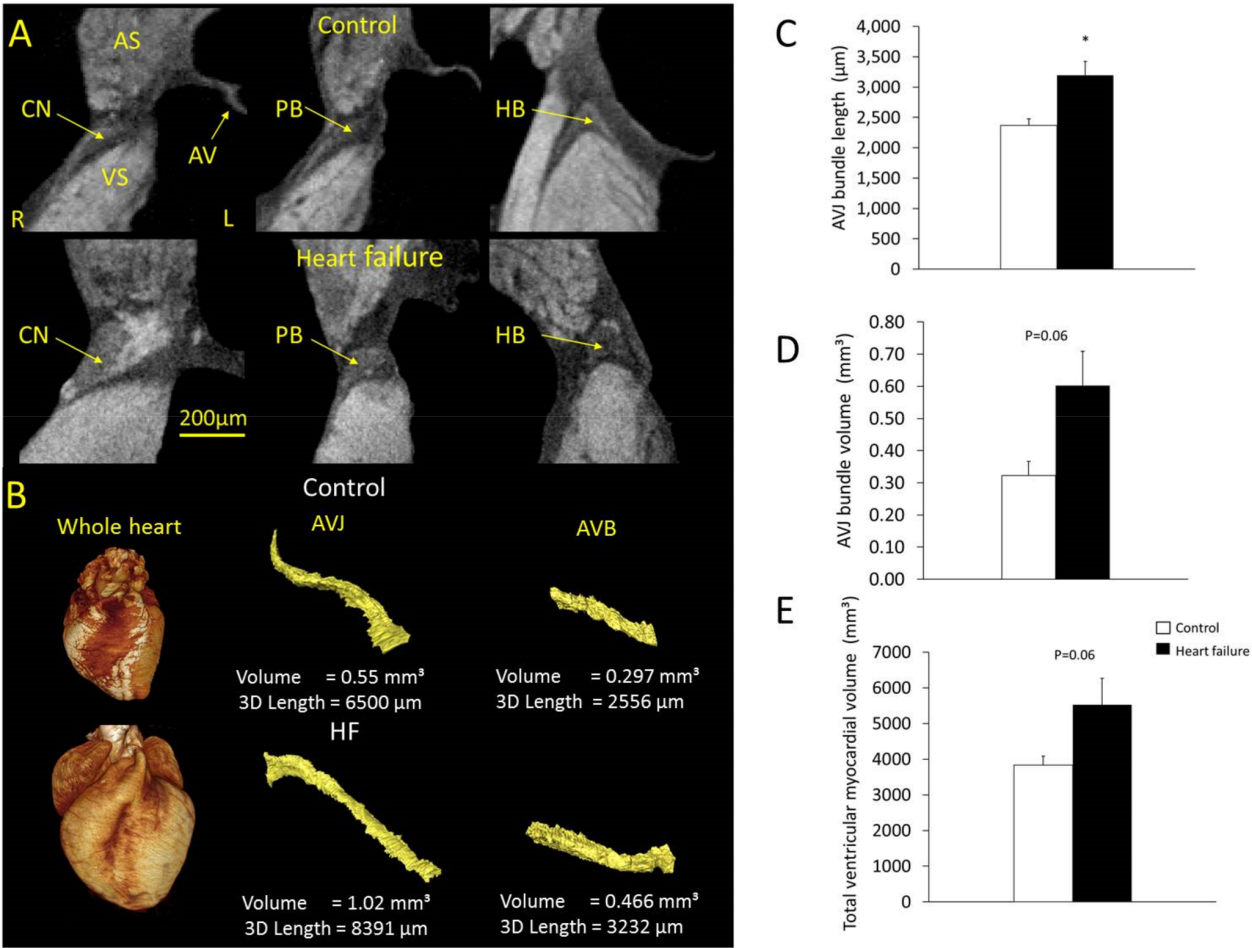
Three-dimensional anatomy of the AVJ demonstrated using micro-CT. A, transverse micro-CT images of the AVJ in control and heart failure animals. B, representative volume reconstructions of one control and one heart failure whole heart and the corresponding AVJs and atrioventricular bundles segmented from micro-CT data, at the same scale for control and heart failure. C, D, measurement of atrioventricular bundle long axis (means±SEM; *P<0.05, n = 5) and volume (means±SEM; P = 0.06, n = 5). E, total ventricular myocardial volume (means±SEM; P = 0.06, n = 5). AVB, atrioventricular bundle. White bars, control animals; black bars, heart failure animals.

In a separate sample of the same experimental group micro-CT allowed not only atrioventricular bundle length measurements but also three-dimensional reconstruction and volume quantification. Total ventricular myocardial volume was also measured in the same hearts (Fig 3). Total ventricular myocardial volume increased in heart failure animals compared to control by 44% (3833±253 mm^3^ vs. 5523±747 mm^3^; n = 5, P = 0.06). Atrioventricular bundle length was 2369±110 μm and volume 0.32±0.04 mm^3^ for control animals vs. 3197±226 μm and 0.60±0.11 mm^3^ for heart failure animals (n = 5; P = 0.016 and 0.06, respectively).

### Cell diameter in the AVJ is not affected by heart failure

**Fig 4:**
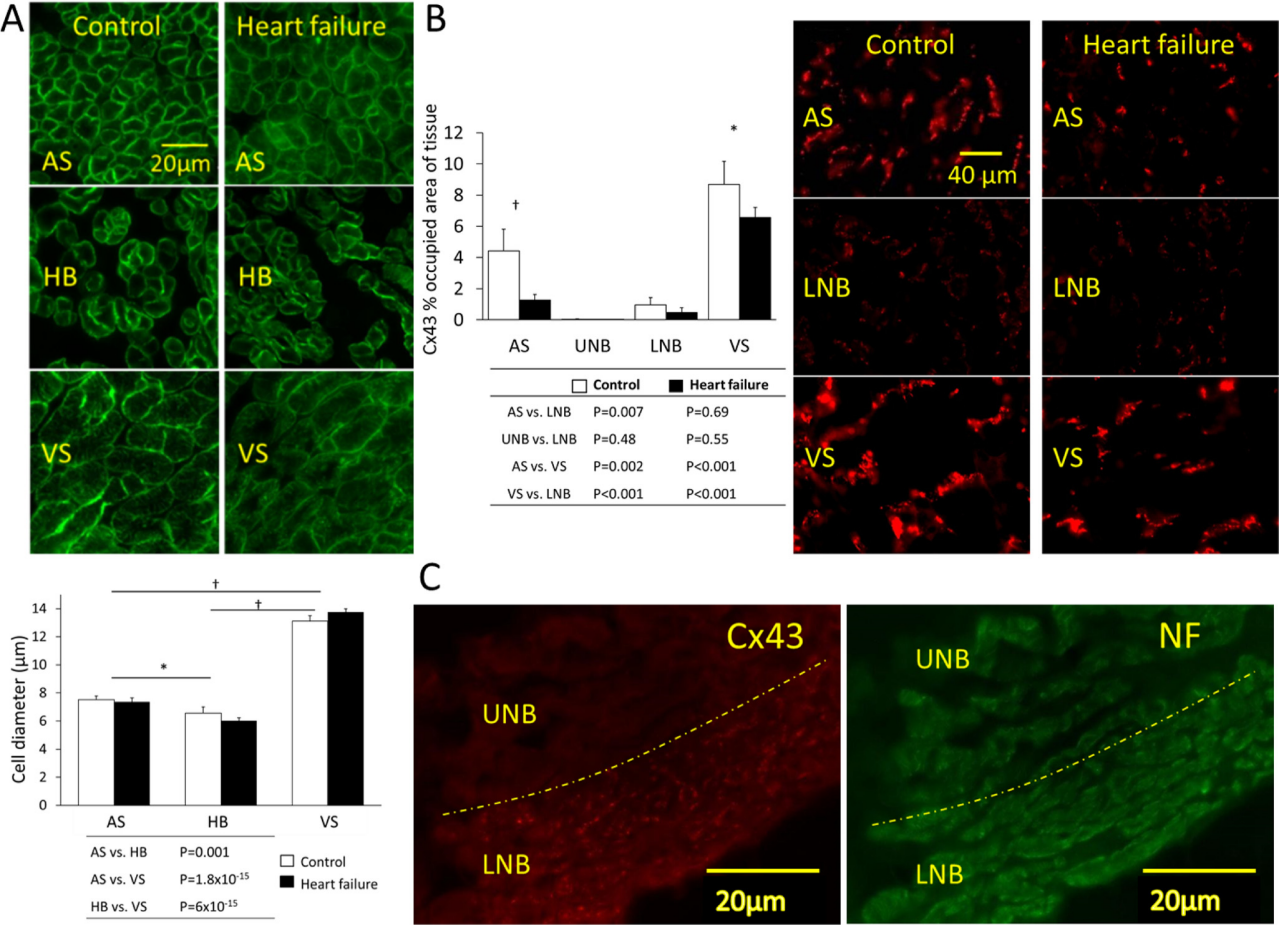
Cell diameter and Cx43 protein expression. A, cell diameter measured using caveolin 3 immunostaining (green signal). Graph shows diameter for cells of the atrial septum, His bundle and ventricular septum (means±SEM, n = 5/6; 2-way ANOVA; *P<0.05, †P<0.001). B, Cx43 protein expression determined by immunohistochemistry. Cx43 (red signal) shown at three regions (atrial septum, lower nodal bundle and ventricular septum). Percentage of tissue area occupied by Cx43 (means±SEM; n = 5/6; 2-way ANOVA; *P<0.05, †P<0.001). C, Cx43 expression in the inferior nodal extension (Cx43, red signal; neurofilament, green signal). The upper nodal bundle is distinguished from the lower bundle because it expresses no Cx43 (separated by the dashed yellow line). AS, atrial septum; L, left; HB, His bundle; LNB, lower nodal bundle; R, right; TT, transitional tissue; UNB, upper nodal bundle; VS, ventricular septum. White bars, control animals; black bars, heart failure animals.

We measured transverse cell diameter of His bundle cells. In all samples, His bundle cells were round, irregular and loosely arranged (Fig 4A). Their morphology was similar to that of atrial myocytes but they were significantly smaller (P = 0.001; Fig 4A). Ventricular myocytes were significantly larger (P = 6×10^−15^). There was no significant increase of cell diameter in heart failure.

### Heart failure causes Cx43 protein downregulation

Immunolabelling for Cx43showed that protein abundance was greatest in ventricular followed by atrial myocardium (Fig 4B). The upper nodal bundle is devoid of Cx43, with very low levels in the lower nodal bundle (Fig 4C). Heart failure was associated with a reduction in Cx43 in the atrial and ventricular septum (n = 5 control, n = 6 heart failure; P = 0.004 and P = 0.044, respectively; Fig 4B).

### Ion channel signature of the AVJ

**Fig 5:**
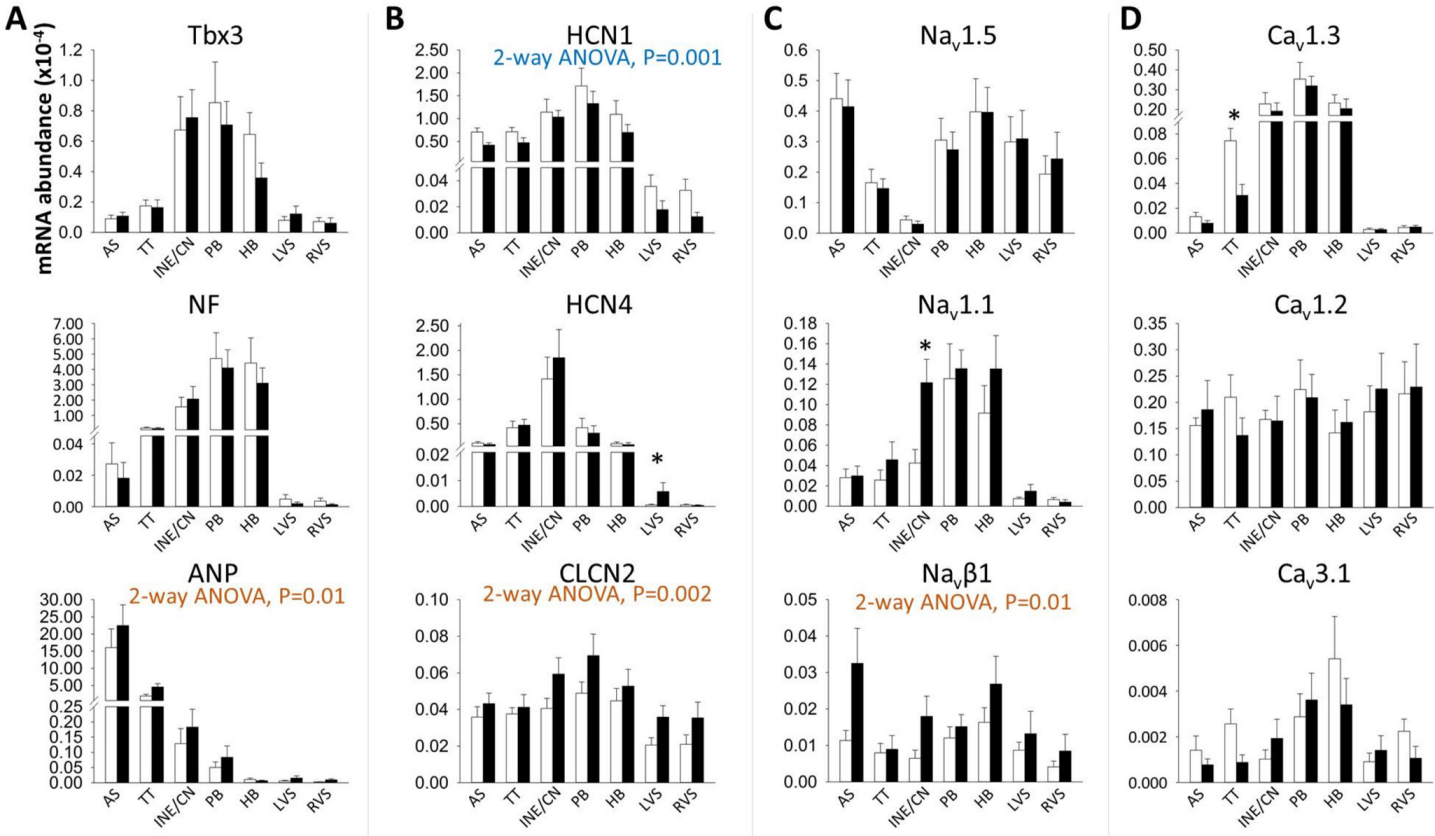
Relative abundance of mRNA for Tbx3, NF, ANP, CLCN2 and *I*_f_, *I*_Na_, *I*_Ca,L_ and *I*_Ca,T_ subunits as measured by RT-PCR. Abundance is relative to that of 28s RNA. Means±SEM, n = 9; 2-way ANOVA used for comparison between control and heart failure animals across all regions (significant P values shown below transcript titles). Limma test used to compare control versus heart failure animals in each region (*P<0.05). AS, atrial septum; HB, His bundle; INE/CN, inferior nodal extension and compact node; LVS, left ventricular septum; PB, penetrating bundle; RVS, right ventricular septum; TT, transitional tissue. White bars, control animals; black bars, heart failure animals.

Differences between control and heart failure samples are discussed in a subsequent section; here we describe regional step-wise variations that apply to both control and heart failure samples (Table 1). The T-box transcription factor 3 (Tbx3) and neurofilament protein (NF) are marker proteins of the cardiac conduction system. Hyperpolarization-activated cyclic nucleotide-gated (HCN) channels carry the funny current (*I*_f_), which is responsible for diastolic depolarization. Tbx3, NF, HCN1 and HCN4 were more abundant in the AVJ compared to atrial and ventricular tissue (Table 1, Fig 5A and 5B).

**Table 1:** Regional variation in ion channel expression.

	Control	P-value	Heart failure	P-value
**Higher in TT vs. AS**	Ca_v_1.3	2.8×10^−3^	HCN4	4.5×10^−2^
**Higher in INE/CN vs. TT**	NF	2.2×10^−2^	NF	1.0×10^−2^
	Ca_v_1.3	3.4×10^−2^	Ca_v_1.3	3.7×10^−3^
	Tbx3	4.3×10^−2^	Tbx3	1.9×10^−2^
			HCN4	4.2×10^−2^
**Higher in PB vs. INE/CN**	Na_v_1.5	1.4×10^−2^	Na_v_1.5	3.6×10^−3^
			TWIK1	1.8×10^−2^
**Higher in LVS vs. HB**	K_ir_2.1	1.4×10^−3^	K_ir_2.1	1.1×10^−2^
	KChIP2	1.2×10^−2^	RYR3	3.3×10^−2^
	SUR2A	1.3×10^−2^	RYR2	4.9×10^−2^
**Higher in RVS vs. LVS**	-	-	KChIP2	2.1×10^−2^

Significant transcriptome changes between adjacent regions in control and heart failure samples (FDR-adjusted Limma test P value). AS, atrial septum; INE, inferior nodal extension; HB, His bundle, PB, penetrating bundle; TT, transitional tissue; LVS, left ventricular septum; RVS, right ventricular septum.

#### Na^+^ and Ca^2+^ channels

The voltage-gated Na^+^ current causes the steep depolarizing upstroke in working myocardium, while pacemaking tissues rely on *I*_Ca,L_ for the action potential upstroke [28,29]. Consistent with this, the main Na^+^ channel isoform Na_v_1.5 was weakly expressed in the AVJ, while Ca_v_1.3 was preferentially expressed in the AVJ compared to atrial and ventricular myocardium (Table 1, Fig 5C and 5D).

#### Voltage-activated K^+^ channels

**Fig 6:**
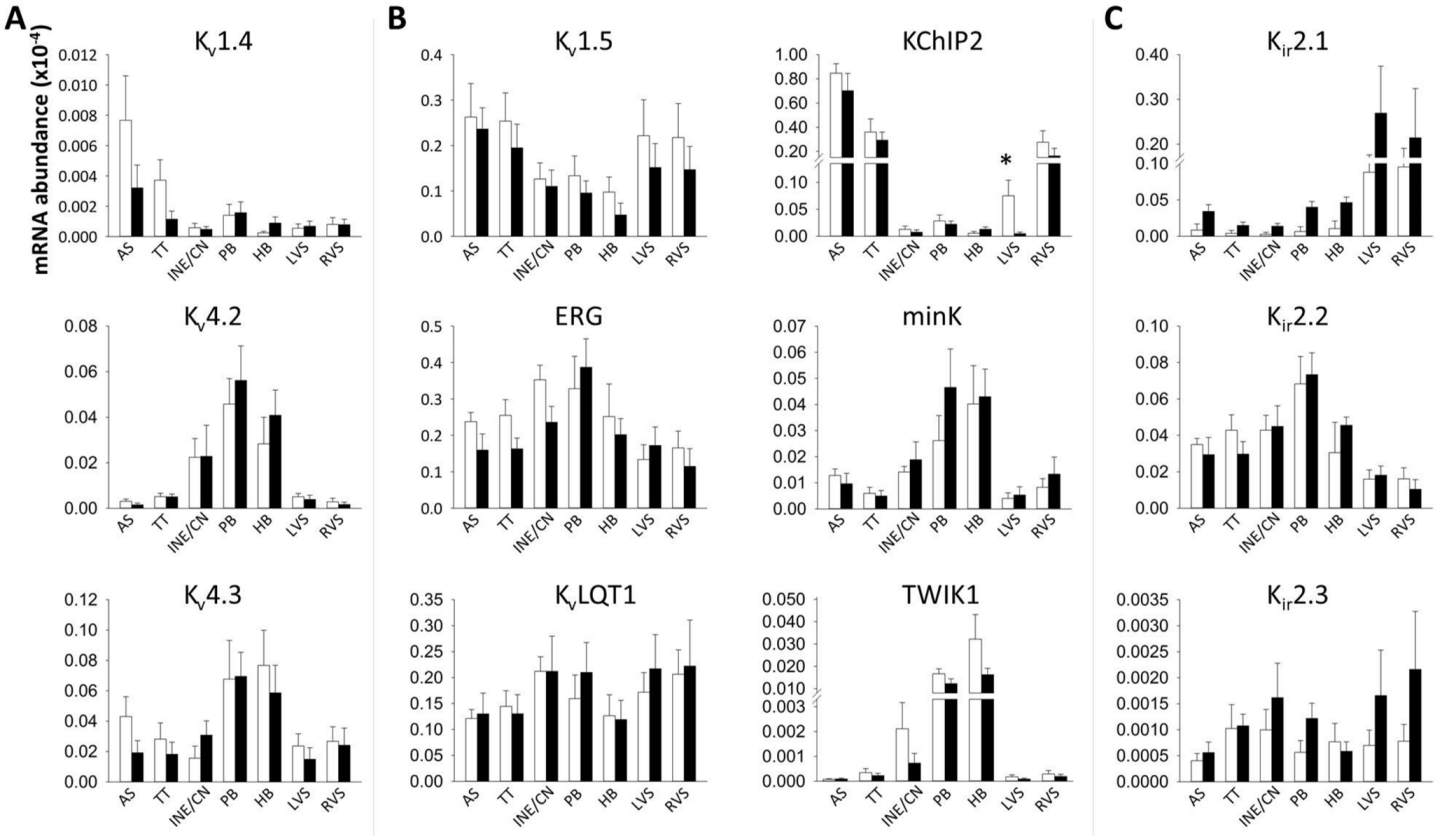
Relative abundance of mRNA for *I*_to_, *I*_K,ur_, *I*_K,r_, *I*_K,s_, *I*_K1_ and TWIK1 α- and β-subunits as measured by RT-PCR. Abundance is relative to that of 28s RNA. Means±SEM, n = 9; *P<0.05 for control animals versus heart failure animals by Limma test. AS, atrial septum; HB, His bundle; INE/CN, inferior nodal extension and compact node; LVS, left ventricular septum; PB, penetrating bundle; RVS, right ventricular septum; TT, transitional tissue; White bars, control animals; black bars, heart failure animals.

K_v_4.2 and K_v_4.3 carry the fast recovering *I*_to_ current (*I*_to,f_) while K_v_1.4 carries the slow-recovering current (*I*_to,s_). K_v_4.2 was more highly expressed in AVJ tissues compared to ventricular and atrial myocardium (Fig 6A). Expression of the K_v_ β-subunit KChIP2 was lower in the AVJ compared to all other regions, while the β-subunit minK, was more prevalent in the penetrating and His bundles compared to ventricular and atrial septum (Fig 6B).

#### Inward rectifying K^+^ channels

**Fig 7:**
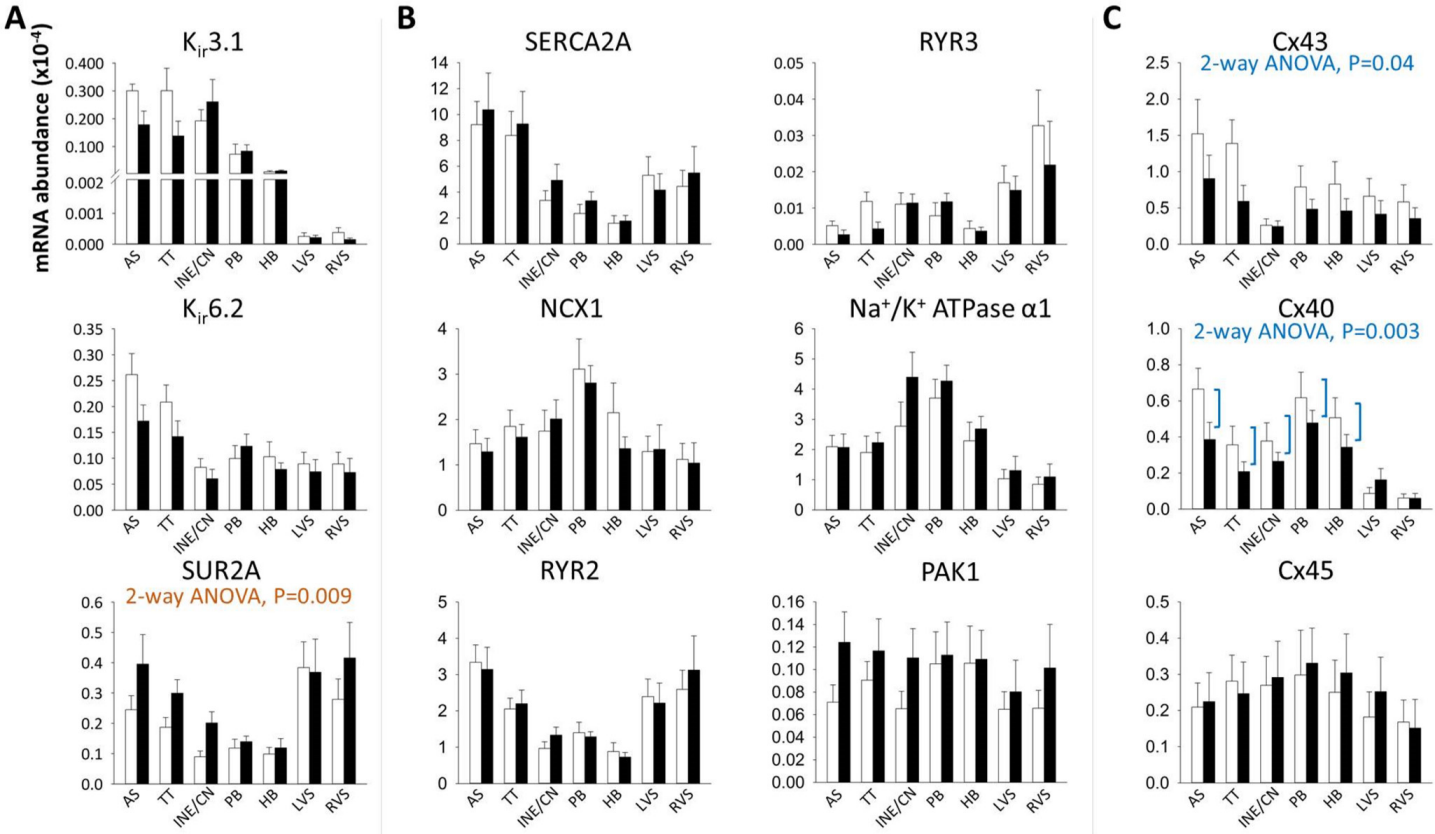
Relative abundance of mRNA for *I*_K,ACh_ and *I*_K,ATP_, transcripts involved in Ca^2+^-handling, PAK1 and connexins as measured by RT-PCR. Abundance is relative to that of 28s RNA. Means±SEM, n = 9. 2-way ANOVA used for comparison between control and heart failure animals across all regions (significant P values shown below transcript titles). AS, atrial septum; HB, His bundle; INE/CN, inferior nodal extension and compact node; LVS, left ventricular septum; PB, penetrating bundle; RVS, right ventricular septum; TT, transitional tissue. White bars, control animals; black bars, heart failure animals.

The background inward rectifier K^+^ current *I*_K,1_ generates the resting membrane potential in myocardial cells. Consistent with this role, the encoding K_ir_2.1 mRNA transcript was significantly more prevalent in ventricular tissue compared to all other regions (Table 1 and Fig 6C). K_ir_2.2 was more abundant in the penetrating and His bundles compared to ventricular myocardium (Fig 6C). TWIK1 is a weakly inward rectifying K^+^ channel, which was also preferentially expressed in the penetrating and His bundles compared to ventricular and atrial tissue (Fig 6B). K_ir_3.1, encoding the acetylcholine-activated current (*I*_K,ACh_), showed preferential expression in the atrial and AVJ tissues compared to the ventricle (Fig 7A).

#### Intracellular Ca^2+^ handling-proteins

SERCA2A levels were significantly lower in the AVJ compared to atrial and ventricular muscle which perhaps reflects the lack of contractile function in the AVJ (Fig 7B). RYR2 and RYR3 were also differentially expressed with higher expression in the ventricle compared to the AVJ (Table 1 and Fig 7B). The Na^+^/K^+^ ATPase α1 transcript was more abundant in the AVJ compared to all other regions (Fig 7B).

### Heart failure causes atrioventricular remodelling

**Fig 8:**
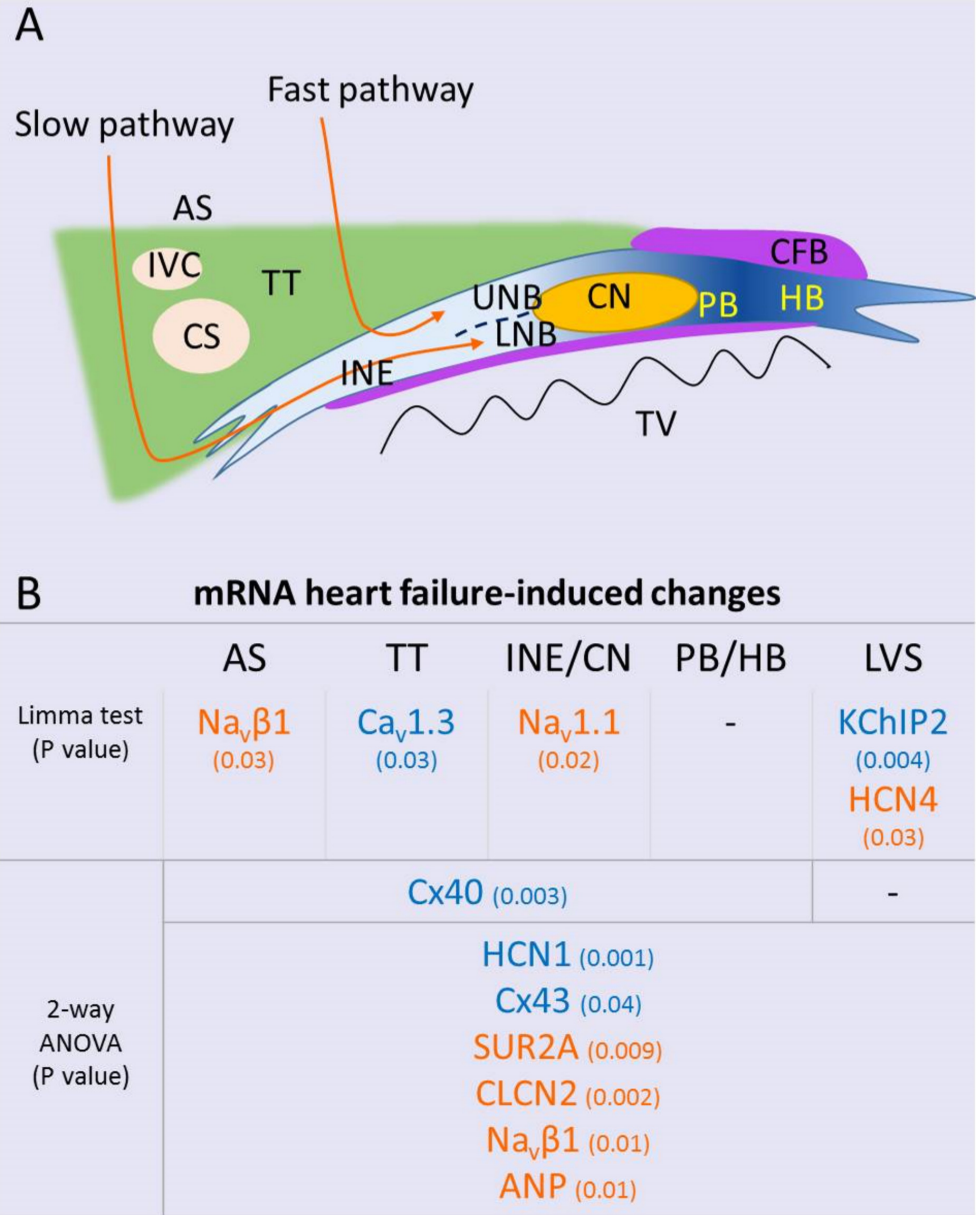
Heart failure-induced mRNA changes. A, schematic of the AVJ. B, summary of heart failure-induced mRNA changes across the AVJ and septal tissues (orange, upregulation; blue, downregulation). AS, atrial septum; AVJ, atrioventricular junction; CFB, central fibrous body; CS, coronary sinus; HB, His bundle; IVC, inferior vena cava; L, left; LNB, lower nodal bundle; LVS, left ventricular septum; PB, penetrating bundle; R, right; TT, transitional tissue; TV, tricuspid valve; UNB, upper nodal bundle.

Heart failure-induced gene expression changes are summarised in Fig 8. Atrial natriuretic peptide (ANP) is a marker of heart failure.[12] ANP mRNA showed upregulation across all areas with heart failure (2-way ANOVA, P = 0.03; Fig 5A). HCN1 showed an overall significant downregulation in heart failure animals (2-way ANOVA, P = 0.001; Fig 5B), while HCN4 was upregulated in the left ventricular septum (Limma test, P = 003; Fig 5B). Chloride channel-2 (CLCN2) is involved in depolarization of the resting membrane potential in conditions of stress. It was significantly upregulated in heart failure tissues (2-way ANOVA, P = 0.002; Fig 5B).

Heart failure caused significant upregulation of the neuronal Na^+^ channel isoform Na_v_1.1 in the inferior nodal extension (Limma test, P = 0.02) and the β-subunit Na_v_β1 (2-way ANOVA, P = 0.01; Fig 5C). Na_v_β1 upregulation was most pronounced in the atrial septum (Limma test, P = 0.03; Fig 5C). Heart failure caused downregulation of Ca_v_1.3 in the transitional tissue (Limma test, P = 0.03; Fig 5D).

Left ventricular septal KChIP2 transcript levels were significantly lower compared to the right ventricular septum (Limma test, P = 0.004; Fig 6B). This has been observed previously [20]. Heart failure caused further suppression of left septal KChiP2 expression (Fig 6B). The adenosine triphosphate (ATP)-sensitive K^+^ current (*I*_K,ATP_) is activated in hypoxic conditions. It is encoded by the pore-forming K_ir_6.2 subunit and the ATP-binding β subunit SUR2A. K_ir_6.2 mRNA was significantly more prevalent in the atrial septum and transitional tissue and decreased in these areas with heart failure (2-way ANOVA, P = 0.07; Fig 7A). In contrast, a significant upregulation in SUR2A mRNA levels was observed (2-way ANOVA, P = 0.009; Fig 7A).

Cx43 mRNA expression was reduced in heart failure across all regions (2-way ANOVA, P = 0.04; Fig 7C). Cx40 was also significantly reduced in heart failure in atrial and AVJ tissues (Fig 7C).

## Discussion

Heart failure leads to increased total ventricular myocardial volume and enlargement of the AVJ (Figs 2 and 3). Most of the heart failure-induced mRNA changes affecting regions of the AVJ also affected the surrounding atrial and ventricular septum in a similar manner. mRNAs for ANP, CLCN2 and Na_v_β1 were increased with heart failure in all regions (Fig 5), while mRNAs for Cx43 and HCN1 were decreased (Figs 5 and 7). Some changes were specific to the AVJ; the downregulation in Ca_v_1.3 mRNA in the transitional tissue and the upregulation of Na_v_1.1 mRNA in the inferior nodal extension/compact node (Fig 5). Cx40 was significantly downregulated in the atrial septum and AVJ tissues but not in the ventricular septum (Fig 7C). KChIP2 downregulation in response to heart failure was seen in the left ventricular septum only (Fig 6B). We suggest that these anatomical and molecular changes may contribute to prolongation of the PR interval.

### Anatomical remodelling of the AVJ in heart failure

Although histological analysis offers good tissue characterisation [16,30,31], recent studies have demonstrated the use of micro-CT for imaging the cardiac conduction system, providing faster, non-destructive anatomical imaging in any plane [21,23]. The Amira ‘3D measure’ tool used in micro-CT length measurements is more accurate compared to histology because it accounts for changes in curvature. In independent samples from the same experimental group, a 26% elongation of the atrioventricular bundle was observed with histological methods compared to 35% using micro-CT. A longer path of travel is expected to result in delayed conduction.

In addition, micro-CT resolved the three-dimensional AVJ anatomy revealing an 87.5% volume increase in the atrioventricular bundle. Total ventricular myocardial volume increased by 44%. In-vivo echocardiography showed left ventricular dilatation with a 43% increase in left ventricular internal diameter in end-diastole. It is therefore likely that anatomical remodelling in the AVJ parallels that of the entire heart. A follow-up study with micro-CT would likely need a larger sample size to investigate AVJ enlargement relative to total heart volume. The ratio of AVJ volume/total ventricular myocardial volume was 1x10^-4^ and not significantly different between control and heart failure samples. Transverse cell diameter in the His bundle, atrial septum and ventricular septum was unchanged.

### Ion channel signature of the AVJ

#### Na^+^ and Ca^2+^ channels

Na_v_1.1 was more abundant in all areas of the AVJ compared to ventricular septum (Fig 5C). This isoform switch from Na_v_1.5 to Na_v_1.1 in the AVJ (Fig 5C) has been observed previously [20,32]. Ca_v_1.2 is the most abundant α_1_-subunit in human myocardium. Cav1.3, an alternative L-type channel Ca^2+^ isoform, is more abundant in the AVJ (Table 1 and Fig 5D), where it contributes to the nodal action potential upstroke [28,33,34]. Ca_v_3.1 was more abundant in the AVJ compared to the working myocardium (Fig 5D), supporting the role of *I*_Ca,T_ in diastolic depolarization.

#### Voltage-activated K^+^ channels

Similar to previous reports in the rabbit [20] and human [32] both K_v_4.2 and K_v_4.3 were expressed in the AVJ, which is in keeping with the presence of *I*_to_ in this area (Fig 6A) [35]. KChIP2 is involved in trafficking of K_v_4 subunits to the cell surface membrane [36] and was poorly expressed in the AVJ (Fig 6B). The *I*_K,s_ β-subunit minK was prominent in the AVJ similar to an earlier report [37]. The significance of this is unclear, since *I*_K,s_ is thought to play only a minor role in atrioventricular node repolarization.

#### Inward rectifying K^+^ channels

Consistent with previous reports [20,31,32] the *I*_K,1_ subunit K_ir_2.1 showed strong presence in the ventricle (Table 1 and Fig 6C), where it maintains a negative resting potential. K_ir_2.2 was significantly more abundant in the penetrating and His bundles compared to ventricular septum (Fig 6C). The twin-pore K^+^ channel TWIK1 was similarly more abundant in the penetrating and His bundles (Fig 6B). K_ir_3.1 predominates in the atria and AVJ (Fig 7A), where it is responsible for tight parasympathetic control. This result is consistent with previous studies in the human [31,32].

### Ion channel and connexin remodelling in heart failure

A previous study in the rat AVJ reported downregulation of HCN4 protein expression in ischaemic cardiomyopathy [38]. This model also displayed prolongation of the PR interval [38]. Three studies have assessed remodelling of the SAN in heart failure [8–10]. Verkerk et al. [8] reported reduction in SAN *I*_K,s_ alongside *I*_f_ in a rabbit model of heart failure, while *I*_to_, *I*_K,ur_, *I*_K,r_, *I*_Ca,L_, *I*_Ca,T_ and *I*_NaCa_ were unchanged. Zicha et al. [9] showed reduced SAN automaticity and downregulation of HCN4 and HCN2 in the SAN (HCN1 is not expressed in canine SAN). Yanni et al. [10] found upregulation of SAN Na_v_1.1, ERG, K_v_LQT1, K_ir_6.2, TWIK1, RYR2 and Cx45 mRNAs in a rat model of ischaemic cardiomyopathy. In a canine model of pacing-induced heart failure, Purkinje cells were found to have reduced *I*_to_ density and slowed *I*_Ca,L_ inactivation at positive potentials [39].

In this study we found region-specific transcriptome changes with a 59% decrease in Ca_v_1.3 in the transitional tissue and a 190% increase in Na_v_1.1 in the inferior nodal extension. Cx40 was downregulated by 34% in the atrial myocardium and AVJ. Changes affecting all regions (including adjacent atrial and ventricular myocardium) were: a decrease in HCN1 (36%) and Cx43 (37%) and an increase in SUR2A (47%), CLCN2 (40%) and Na_v_β1 (89%).

Cx43 expression was reduced both at the mRNA and protein levels (Figs 4B and 7C) in keeping with a previous report in the human failing left ventricle [40].

We hypothesise that a decrease of HCN1 and Ca_v_1.3 could be responsible for a slowing of atrioventricular node conduction. Both channels carry inward i.e. excitatory current. Knockout of an HCN channel (HCN4) in the mouse causes atrioventricular block [41]. Knockout of Ca_v_1.3 in the mouse causes atrioventricular block and reduced automaticity [33,34]. Downregulation of the connexins is of course expected to slow AV node conduction. On the other hand, however, the upregulation of CLCN2, Na_v_1.1 and Na_v_β1 (inward current channel subunits) is not expected to slow atrioventricular node conduction. We therefore hypothesise that these changes may be compensatory.

Heart failure was associated with a 9-fold upregulation of HCN4 in the ventricular septum. HCN4 channel mRNA upregulation has been described in human failing atrial and ventricular myocardium [42] and it may be implicated in ventricular arrhythmogenesis.

## Conclusion

This study adds to our understanding of the pathophysiology of atrioventricular block in heart failure by identifying anatomical enlargement of the atrioventricular conduction tissues, which is in keeping with global heart enlargement, and remodelling in *I*_f_ and *I*_Ca_ channels, as well as connexins. Anatomical enlargement is expected to lead to prolongation of the PR interval because of a longer path of travel. In addition, changes at the mRNA level, taken together, may also contribute to prolongation of the PR interval. PR interval prolongation may therefore be related with both heart enlargement and/or molecular remodelling. Future studies are needed to elucidate the mechanisms by which heart failure induces these changes. Developing clinical modalities for fast and high-resolution imaging of the cardiac conduction system may help guide personalised medical and device therapy for heart failure.

## Study Limitations

We studied heart failure-induced transcriptome changes that occur in the regions in and immediately surrounding the atrioventricular node. The physiological importance of these changes is likely to differ in different regions of the heart. Unravelling the precise anatomical site of atrioventricular block requires invasive electrophysiological measurements in a future study. The phosphorylated state of gap junctions can affect their conductance. Future studies are needed to investigate the effect of heart failure on phosphorylated Cx43 in the AVJ.

We did not observe higher degree atrioventricular block (second or third) in any animals. It is unknown whether rabbits are protected from progression to higher degree atrioventricular block in heart failure, thus representing a species difference to the human. The rabbit AVJ is smaller and has only one inferior (or posterior) extension. It also has the capability to conduct faster heart rates compared to the human. No animal model satisfactorily reproduces the features of human heart failure, which is a multifactorial process involving hypertension, ischaemic heart disease and diabetes. In addition, no model reproduces the long time-frame involved in the development of human heart failure. Our model of heart failure has advantages in that it is physiological, preserves native cardiac conduction and induces a gradual decline of left ventricular function over time.

## Supporting Information

S1 Checklist “The ARRIVE Guidelines Checklist” for reporting animal data in this manuscript. (DOCX)

S1 File Supplementary Methods. Immunohistochemistry and Masson’s trichrome. (DOCX)

S1 Table Details of primers used in RT-PCR. (DOCX)
