# Supplementary material for: Congestive Heart Failure Leads to Prolongation of the PR Interval and Atrioventricular Junction Enlargement and Ion Channel Remodelling in the Rabbit
Source: PLoS One. 2015 Oct 28;10(10):e0141452. doi: 10.1371/journal.pone.0141452 (PMC4624927; doi:10.1371/journal.pone.0141452)

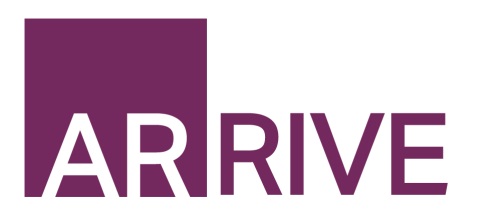


The ARRIVE Guidelines Checklist

Animal Research: Reporting In Vivo Experiments

Carol Kilkenny^1^, William J Browne^2^, Innes C Cuthill^3^, Michael Emerson^4^ and Douglas G Altman^5^

*^1^The National Centre for the Replacement, Refinement and Reduction of Animals in Research, London, UK, ^2^School of Veterinary Science, University of Bristol, Bristol, UK, ^3^School of Biological Sciences, University of Bristol, Bristol, UK, ^4^National Heart and Lung Institute, Imperial College London, UK, ^5^Centre for Statistics in Medicine, University of Oxford, Oxford, UK.*

|  | | ITEM | RECOMMENDATION | Section/ Paragraph |
| --- | --- | --- | --- | --- |
| 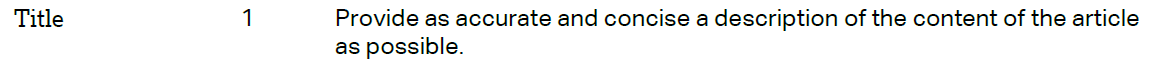 | | | Title |  |
| 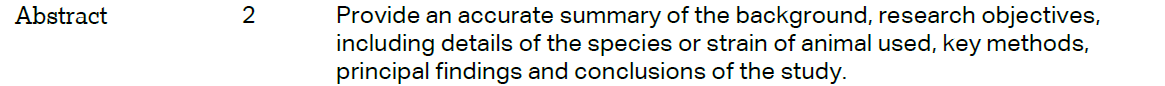 | | | Abstract |  |
| INTRODUCTION | | |  |  |
| 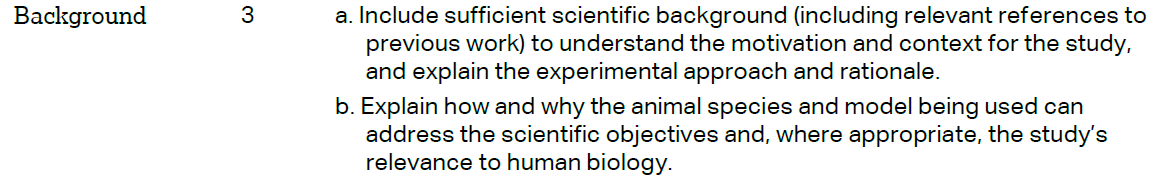 | | | Introduction  Introduction Paragraph 3 |  |
| 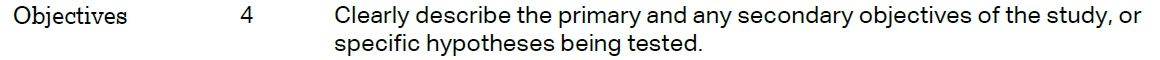 | | | Methods Paragraph 1 |  |
| METHODS | | |  |  |
| 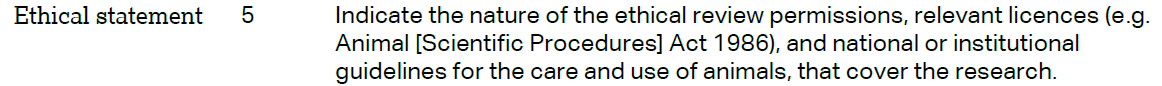 | | | Methods Paragraph 2 |  |
| 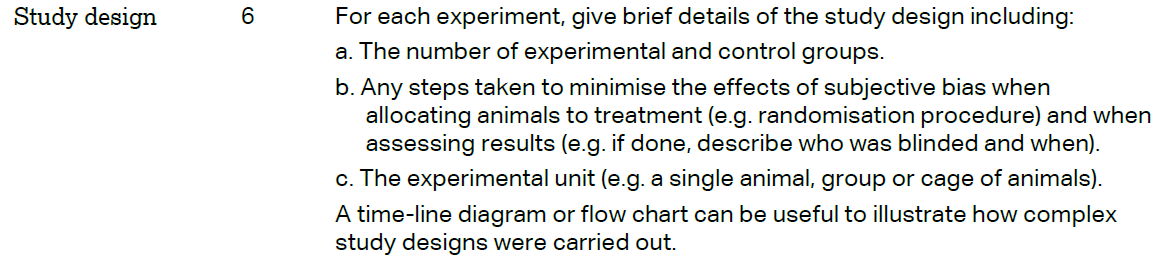 | | | Methods Paragraph 1  Methods Paragraph 2  Methods Paragraph 2 |  |
| 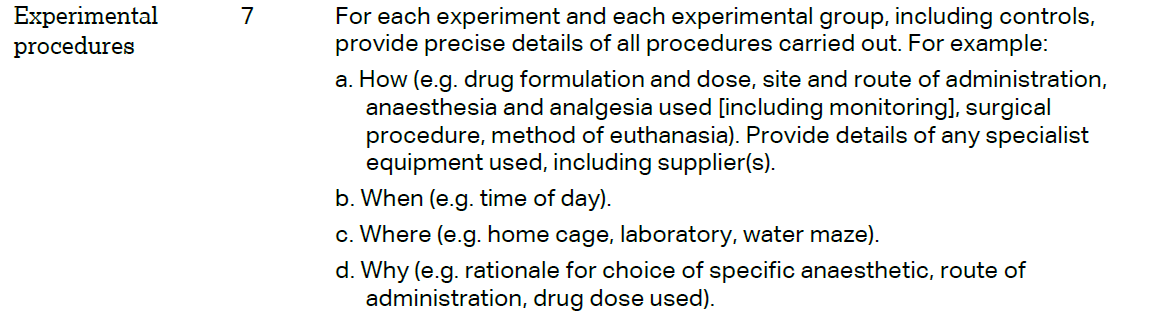 | | | Methods Paragraph 3  Methods Paragraph 3  Methods Paragraph 3  Methods Paragraph 3 |  |
| 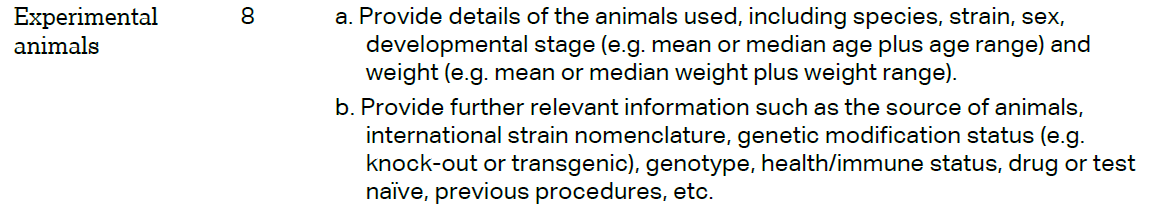 | | | Methods Paragraph 2  Methods Paragraph 2 |  |

The ARRIVE guidelines. Originally published in *PLoS Biology*, June 2010^1^

| 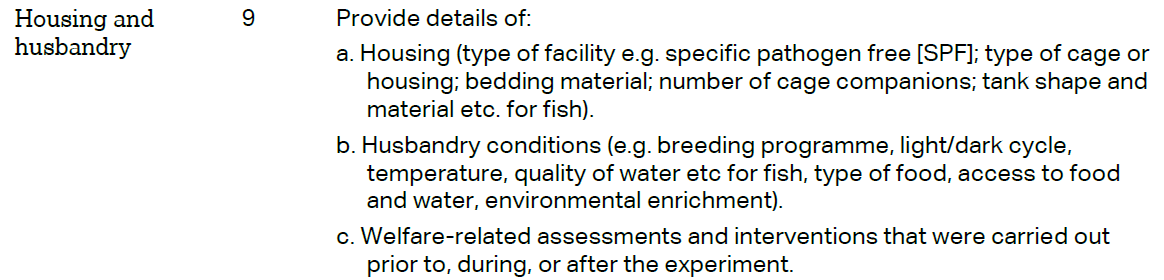 | Methods Paragraph 2  Methods Paragraph 2  Methods Paragraph 2 | |
| --- | --- | --- |
| 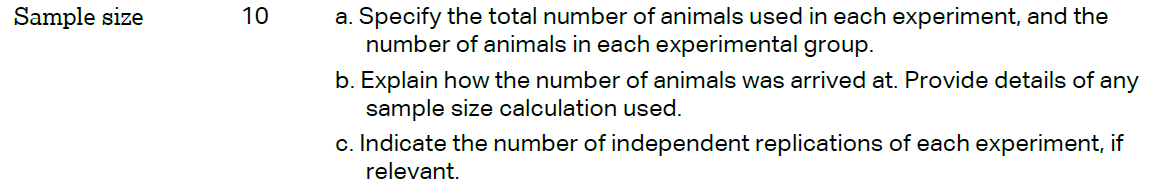 | Methods Paragraph 1  Methods Paragraph 1  N/A | |
| 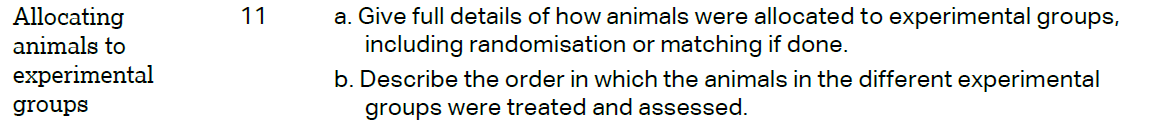 | Methods Paragraph 2  Methods Paragraph 3 | |
| 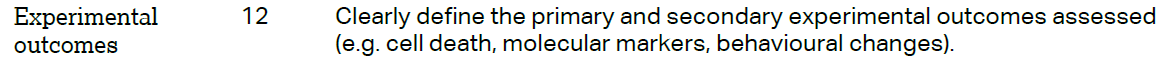 | Methods Paragraph 1 | |
| 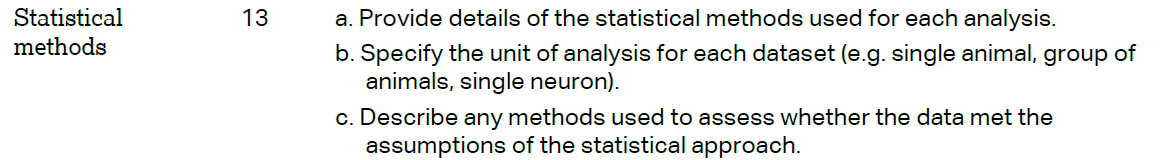 | Methods Paragraph 1 Methods Paragraph 8 | |
| RESULTS |  | |
| 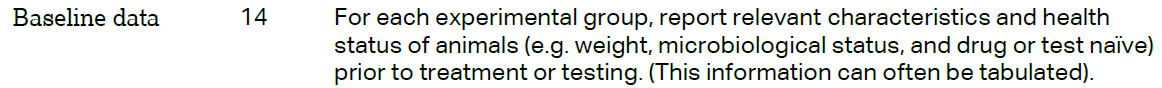 | Methods Paragraph 2  Results Paragraph 1  Figure 1 | |
| 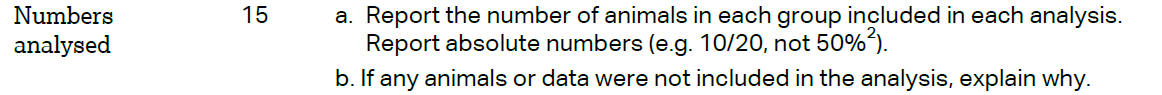 | Methods Paragraph 1, Results and Figures  N/A | |
| 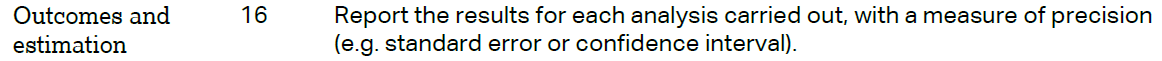 | Results Paragraphs 1-13 | |
| 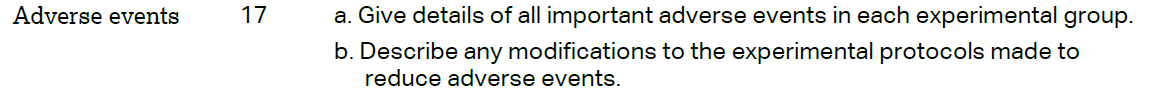 | Methods Paragraph 3  Methods Paragraph 3 | |
| DISCUSSION |  | |
| 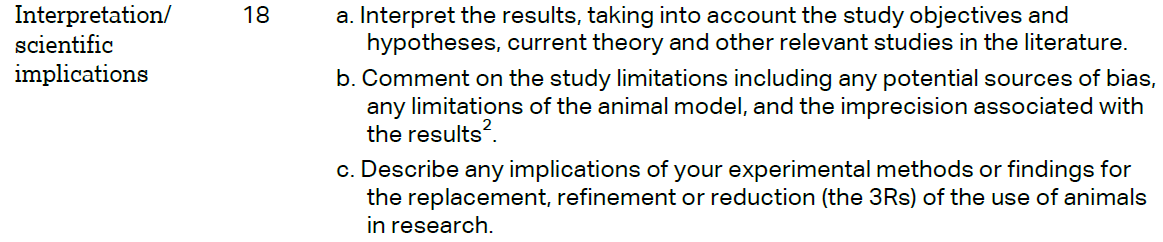 | Throughout  Discussion Paragraph 11  Discussion Paragraph 11 | |
| 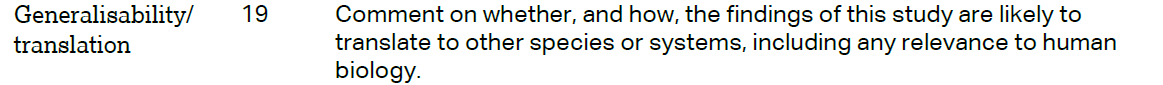 | Conclusion | |
| 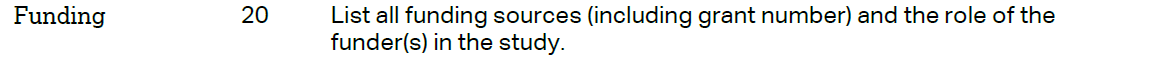 | | Details submitted online |


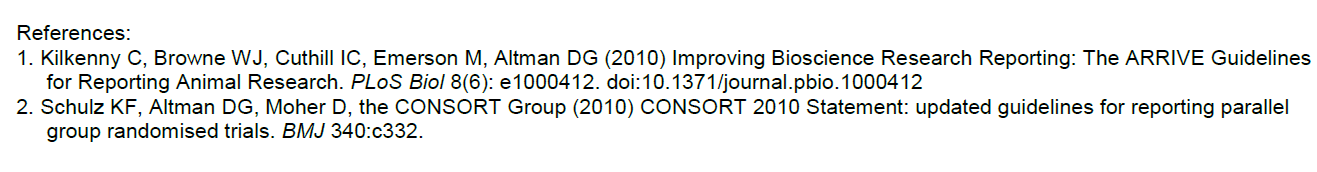

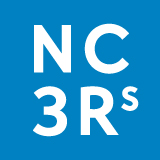

Supplement: S1 Checklist — (DOCX) [file pone.0141452.s001.docx]
