# Supplementary material for: Congestive Heart Failure Leads to Prolongation of the PR Interval and Atrioventricular Junction Enlargement and Ion Channel Remodelling in the Rabbit
Source: PLoS One. 2015 Oct 28;10(10):e0141452. doi: 10.1371/journal.pone.0141452 (PMC4624927; doi:10.1371/journal.pone.0141452)
Supplement: S1 File — (DOCX) [file pone.0141452.s002.docx]

**S1 File. Immunohistochemistry and Masson’s trichrome methods.**

AVJ tissues were dissected in Tyrode’s solution immediately after termination, frozen in isopentane above liquid N and stored at -80°C. They were sectioned at 10 µm, mounted and immersion-fixed in 10% phosphate-buffered formalin solution (PBFS) before rinsing in phosphate-buffered saline (PBS). They were permeabilised with 0.1% triton X-100 for 30 min and treated for 60 minutes with 1% bovine serum albumin. Primary antibodies applied overnight at 4°C were: mouse monoclonal anti-neurofilament (anti-NF) IgG (1:500; Developmental Studies Hybridoma Bank, 2H3, raised against rat neurofimament 165 kDa), rabbit polyclonal anti-Cx43 IgG (1:400; Sigma, C6219, raised against amino acids 363-382 of human/rat Cx43) and mouse monoclonal anti-caveolin 3 IgG (1:200; BD Transduction Laboratories, 610420, raised against amino acid 3-24 of rat caveolin 3). Use of these antibodies has previously been validated [[1](#_ENREF_1),[2](#_ENREF_2)]. Samples were rinsed in PBS. Secondary antibodies used were: donkey anti-rabbit conjugated to Cy3 (1:100, Millipore, AP182C) and donkey anti-mouse conjugated to FITC (1:100, Millipore, AP192F). Sections were rinsed and mounted in Vectashield mounting medium (Vector Labs).

An epifluorescence Olympus BX51 microscope was used for imaging immunolabelled sections. For Cx43 protein quantification images were taken with a 40× oil immersion lens (numeric aperture, 1.4). Total tissue area was measured (excluding gaps) and a background subtraction algorithm applied. After reviewing all slides, threshold intensity was set at 900-4095 to ensure that Cx43 labelled areas were being correctly identified. Percentage area occupied by Cx43 and integrated density of fluorescence were measured. All images were acquired at 50 ms exposure time and the full range of intensity. Caveolin 3 immunolabelled sections were imaged using the same lens. Each image encompassed an area of 9265 µm^2^. Image analysis was performed using ImageJ (NIH). We assumed that most cells would be cut transversely, that is in the plane of their minimum dimension. The minimum diameter of the cell outline was measured, to compensate for any obliquity of sectioning. Cell diameter was averaged for 10-15 cells per image.

**Masson’s trichrome**

10 µm cryosections were fixed from frozen in preservative Bouin's solution (Sigma) overnight and washed three times in 70% ethanol (10 min each wash). Stains were freshly made and filtered [[3](#_ENREF_3)]. A two-stage nuclear stain was used (celestine blue and Cole's haematoxylin). Acid fuchsin was used to stain the cytoplasm and methyl blue for collagen. Two differentiators were used (phosphomolybdic acid and 1% acetic acid). After staining, sections were dehydrated through 70%, 95% and 100% ethanol for 5 min each and cleared in Histoclear solution. Finally, slides were mounted with glycerol gelatin aqueous mounting medium (Sigma). Sections were examined with a 20× lens (numeric aperture, 0.8) using a 3D Histech Panoramic 250 Flash II slide scanner.

**References**

1. Dobrzynski H, Li J, Tellez J, Greener ID, Nikolski VP, et al. Computer three-dimensional reconstruction of the sinoatrial node. Circulation 2005; 111: 846-854.

2. Ripplinger CM, Li W, Hadley J, Chen J, Rothenberg F, et al. Enhanced transmural fiber rotation and connexin 43 heterogeneity are associated with an increased upper limit of vulnerability in a transgenic rabbit model of human hypertrophic cardiomyopathy. Circ Res 2007; 101: 1049-1057.

3. Suvarna K, Layton C, Bancroft JD. Bancroft's theory and practice of histological techniques. xiv, 637 pages. 2013.
