# Supplementary material for: Congestive Heart Failure Leads to Prolongation of the PR Interval and Atrioventricular Junction Enlargement and Ion Channel Remodelling in the Rabbit
Source: PLoS One. 2015 Oct 28;10(10):e0141452. doi: 10.1371/journal.pone.0141452 (PMC4624927; doi:10.1371/journal.pone.0141452)
Supplement: S1 Table — (DOCX) [file pone.0141452.s003.docx]

**S1 Table. Details of primers used in RT-PCR.**

| **Gene target** | **Accession number** | **Primer sequence 5´-3´** | **Product size (bp)** | **Optimal concentration (nM)** |
| --- | --- | --- | --- | --- |
| 28s | AF460236 | gttgttgccatggtaatcctgctcagtacg  tctgacttagaggcgttcagtcataatccc | 133 | 300/300 |
| GAPDH | L23961 | gattccacccacggcaagt  CGCTCCTGGAAGATGGTGAT | 92 | 900/900 |
| Tbx3 | XM_002719784 | cacctgaggcctctcaagac  ccttcccgacttggtaatga | 135 | 300/300 |
| ANP | M12046 | ggaaccgagaggaaggaaga  ccagaatgccagacagaagag | 111 | 50/50 |
| NF | Z47378 | ggtgagcaggaagaaggaga  actggtgattttggcacagg | 176 | 900/50 |
| Cx40 | DQ242483 | gaggaagtgaacggcagga  caggaagatccgtagagcag | 129 | 300/300 |
| Cx43 | DQ242486 | cgtttacacctgcaaaagagacc  ccaccagcatgaagataatgaaga | 98 | 900/300 |
| Cx45 | DQ242487 | atgtacctgggctatgccattc  ggtcctcttcatggtcctcttct | 145 | 300/900 |
| HCN4 | AB022927 | gctgtcctgtcgtcttgtttctc  cggttattgttggtggtgtatctc | 74 | 900/900 |
| HCN1 | AF168122 | tcaggttttattgggatttaataatgct  tggtgttgttgtctgttctgtaaaga | 101 | 300/900 |
| Na_v_1.1 | DQ242485 | gcaggccaatgtccagaa  agcccagctgaaggtatcaa | 90 | 300/900 |
| Na_v_1.5 | DQ242484 | ttgtggacctgggcaatg  tcttgaggcctgcaatgact | 87 | 300/900 |
| Na_v_β1 | P53788 | cagaagggcactgaggagtt  tggtgatgaagatggacagg | 148 | 900/900 |
| Ca_v_1.3 | DQ220009 | aggtagagcggtccgagtcctt  ctctgcttctcgctgtgtttgtt | 162 | 300/50 |
| Ca_v_1.2 | X15539 | gagtcaccctcccacgagaa  gcagtgttgttggcattgttg | 101 | 300/900 |
| Ca_v_3.1 | XM_002719106 | ATCTCTTCATCACGGGTGTCATT  CACGAGTTTGAAGACAGACTCCA | 149 | 900/900 |
| K_v_1.4 | AF493544 | tcctcttcattggggtcatc  agctgcgtctgttcctcatt | 286 | 900/300 |
| K_v_4.2 | AF508735 | gttcgctgagtggtgtcttg  ttgtctgctcgttggttttg | 94 | 300/300 |
| K_v_4.3 | AF198445 | cttctaccgcaccggcaagct  tcctggttgttctccgagtcgtt | 186 | 300/300 |

**S1 Table continued.**

| **Gene target** | **Accesion number** | **Primer sequence 5´-3´** | **Product size (bp)** | **Optimal concentration (nM)** | |
| --- | --- | --- | --- | --- | --- |
| minK | L41659 | ctacatccgctcccagaaact  gctggttttcaaggacgtagc | 139 | | 900/900 |
| K_v_1.5 | AF056943 | agctgcttgtgcgtttctttg  agggtgatgaagtaggggaagatg | 103 | | 300/300 |
| ERG | U87513 | ggctatcctggggaagaatg  gtcccggtggatcttgtg | 121 | | 300/900 |
| K_v_LQT1 | AJ291316 | aggagctgatcaccaccctgta  atctgcgtagctgccgaactc | 124 | | 900/900 |
| KChIP2 | DQ218451 | aacaactgcaggagcaaatcaag  ggtgtcaaaggcattgaagagaa | 182 | | 900/300 |
| TWIK1 | AY752982.1 | tcaccgtgtcctgcttcttctt  tagccttctccaggaacgtaatctc | 142 | | 900/900 |
| K_ir_2.1 | AF021138 | tcatcaacgtgggtgagaag  ccaaaacacacagccaaaaa | 140 | | 300/900 |
| K_ir_2.2 | AJ291313 | ttcgtcaagaagaacggaca  aaggccagcgagaagataag | 137 | | 900/900 |
| K_ir_2.3 | P48050 | tgccaacctgagcaacaa  aagagccaggagacgagga | 120 | | 900/900 |
| K_ir_6.2 | AF006262 | tttcctccgccttccttttct  atcatgagccctacgatgttctg | 127 | | 300/900 |
| SUR2A | AF087468 | cgtactccgagagggaactttg  atcctgacggttcatgagtgttt | 88 | | 300/900 |
| Na^+^/K^+^ ATPase α_1_ | AF235024 | ctgtggatcggagcgattct  caccacgccaaggtacagatt | 96 | | 900/900 |
| RYR2 | OCU50465 | gtgttgcctctccacctctc  cctcacctgtggtgtcctct | 80 | | 50/300 |
| RYR3 | X68650 | caccaaagcacagagggaca  aagacgagacgtcgcaggag | 99 | | 900/900 |
| NCX1 | S66652 | tccatgctagagaccatcctgtt  cttggtgtgttctcccaagatg | 143 | | 50/300 |
| SERCA2A | X02814 | tgaaaatgcaattgaagccct  acactctttctgtcctgccga | 75 | | 900/300 |
| PAK1 | XM_002714170 | aaagcacccccgtctttactct  gctttctttagcctccatcac | 96 | | 900/900 |
| CLCN2 | S80361 | CAGGTGATGAGGAAGCAGAAGA  ATGAACTGTCCAAAGCCAGGAG | 121 | | 300/300 |
